# Lipidomic and Transcriptomic Analysis of the Longissimus Muscle of Luchuan and Duroc Pigs

**DOI:** 10.3389/fnut.2021.667622

**Published:** 2021-05-07

**Authors:** Zhiwang Zhang, Qichao Liao, Yu Sun, Tingli Pan, Siqi Liu, Weiwei Miao, Yixing Li, Lei Zhou, Gaoxiao Xu

**Affiliations:** ^1^Teaching and Research Section of Biotechnology, Nanning University, Nanning, China; ^2^State Key Laboratory for Conservation and Utilization of Subtropical Agro-Bioresources, College of Animal Science and Technology, Guangxi University, Nanning, China

**Keywords:** intramuscular fat, lipomics, pork, Luchuan, Duroc

## Abstract

Meat is an essential food, and pork is the largest consumer meat product in China and the world. Intramuscular fat has always been the basis for people to select and judge meat products. Therefore, we selected the Duroc, a western lean pig breed, and the Luchuan, a Chinese obese pig breed, as models, and used the longissimus dorsi muscle for lipidomics testing and transcriptomics sequencing. The purpose of the study was to determine the differences in intramuscular fat between the two breeds and identify the reasons for the differences. We found that the intramuscular fat content of Luchuan pigs was significantly higher than that of Duroc pigs. The triglycerides and diglycerides related to flavor were higher in Luchuan pigs compared to Duroc pigs. This phenotype may be caused by the difference in the expression of key genes in the glycerolipid metabolism signaling pathway.

## Introduction

Pork is one of the largest consumer meat products in the world (1). Global pork production has quadrupled over the past 50 years and it is projected that it will continue to increase over the next three decades (2). In order to satisfy the huge demand, more and more breeding enterprises have chosen pig breeds that grow rapidly. However, with the improvement of living standards, the demand for quality pork is becoming higher and higher. Previous studies found that people prefer pork with more marbling (3, 4). However, fast-growing pig breeds such as Landrace, Large White and Duroc cannot meet this demand.

Duroc is one of the main commercial pig breeds, famous for its fast growth rate and high lean meat rate (5). However, its intramuscular fat (IMF) content is low which may influence the meat quality. Compared with these lean pig breeds, Chinese native breeds have attracted much attention for their excellent meat quality (6). The Luchuan pig is a famous Chinese native black-and-white species with high meat quality, high reproductive performance and a slow growth rate (7). It is mainly distributed in the Luchuan area of Guangxi Province, China. As a typical fatty pig breed, the Luchuan pig has low lean meat rate and strong fat deposition ability, specifically demonstrating high IMF content. Therefore, studying the difference in lipids in the longissimus dorsi muscle of Luchuan and Duroc pigs may provide directions for improving meat quality.

IMF content is an important indicator of pork quality, which is related to tenderness, flavor and juiciness (8, 9). In skeletal muscle, lipids are stored in the cytoplasm of muscle fibers and fat cells (interspersed between fiber bundles) in the form of lipid droplets (10). In the past few years, the research on IMF has attracted more and more attention. Therefore, if we want to improve meat quality and meet market demand, increasing the IMF content will be an essential step.

Driven by high-performance and high-resolution mass spectrometry-based lipidomics research, lipidomics methods have been used to enhance food science research for various purposes (11). Lipidomics has been used in researches on the detection of dairy products (12) and pork components (13). Lipidomics approaches include nuclear magnetic resonance spectroscopy (NMR), gas chromatography mass spectrometry (GC–MS), and liquid chromatography mass spectrometry (14). Compared with the NMR and GC–MS, the combination of liquid spectroscopy and mass spectrometry can perform quantitative and molecular identification, which is more popular with researchers (15).

Many studies has been conducted to determining the relationship among the intramuscular lipid, meat quality and flavor. However, there are few studies on muscle lipidomics in obese and lean pigs. We conducted a lipidomics test on the lean Duroc pig and the obese Luchuan pig to analyze the differences in lipid types and amounts between the two pig breeds. Through the joint analysis of lipidomics and transcriptomics, the causes of differences in lipid content were analyzed and different pathways were revealed. Our study investigated lipid differences among different types of pig breeds, and provides a reference for improving IMF content.

## Materials and Methods

### Animals

Luchuan and Duroc boar pigs (*n* = 6) were used in this study. Duroc pigs were purchased from Guangxi State Farms Yongxin Animal Husbandry Group Co. Ltd. Luchuan pigs were purchased from The Animal Husbandry Research Institute of Guangxi Zhuang Autonomous Region. The breeding conditions of the two pigs are the same (Table 1). All pigs were sacrificed at 180 days of age. Samples were collected and frozen with liquid nitrogen and then stored at −80°C. The protocol was approved by the Committee on the Ethics of Animal Experiments of Guangxi University (GXU2015-003).

**Table 1:** Dietary nutrition levels.

**Nutrition level**	**Content (%)**
Energy (KJ/kg)	11.98
Crude protein (%)	16
Crude fiber (%)	6
Crude ash (%)	7
Calcium (%)	0.6–1.2
Total phosphorus (%)	0.4–1.0
NaCl (%)	0.2–0.8
Lysine (%)	0.8

### Tissue Triglyceride Measurement

Triglyceride content was tested by a tissue/cell triglyceride (TG) kit (16, 17) (Pulilai, Beijing, China). Briefly, tissue (0.1 g) was placed in a centrifuge tube, 1 ml RIPA lysis solution (Solarbio, Beijing, China) was added and steel balls were used to grind with a tissue grinder. Samples were then lysed overnight on a shaker. The lysate was centrifuged at 12,000 RPM for 10 min at 4°C, the supernatant was collected and TG and protein content was measured (16) by microplate reader (Tecan infinite M200 PRO). The TG level was normalized to the protein concentration.

### Western Blot

Tissue was lysed in RIPA lysis buffer containing 1 mM PMSF. The total protein concentration was determined using a BCA protein assay kit (Biyuntian, Shanghai, China). The centrifuged supernatant was boiled and SDS-PAGE electrophoresis was performed (Mini-PROTEAN Tetra System, Bio-Rad), followed by transfer to a PVDF membrane. Subsequently, the primary antibodies anti-slow skeletal myosin heavy chain (1:1,000; ab11083, Abcam, Cambridge, UK), anti-fast skeletal myosin heavy chain (1:1,000; ab51236, Abcam), *β*-tubulin antibody (1:1,000; 2146s, Cell Signaling Technology, Inc., Shanghai, China) were incubated overnight at 4°C. The Image Lab (Universal Hood II, Bio-Rad) was used to detect chemiluminescent signals after the secondary antibody incubation (18).

### Lipidomic Sequencing

The frozen sample was crushed using a mixer mill (MM 400, Retsch, Germany) with a zirconia bead for 1.5 min at 30 Hz. The resulting powder was weighed and 100 mg was extracted overnight with 1.2 ml 70% aqueous methanol at 4°C. Following centrifugation at 12,000 rpm for 10 min, the extracts were filtered (SCAA-104, 0.22 μm pore size; ANPEL, Shanghai, China) before UPLC-MS/MS analysis. The sample extracts were analyzed using an UPLC-ESI-MS/MS system (UPLC, Shim-pack UFLC SHIMADZU CBM30A system, www.shimadzu.com.cn/; MS, Applied Biosystems 4500 Q TRAP, www.appliedbiosystems.com.cn/). LIT and triple quadrupole (QQQ) scans were acquired on a triple quadrupole-linear ion trap mass spectrometer (Q-TRAP), API 4500 Q-TRAP UPLC/MS/MS System, equipped with an ESI Turbo Ion-Spray interface, operating in positive and negative ion mode and controlled by Analyst 1.6.3 software (AB Sciex). The criteria for determining differential metabolites were: Fold change ≥2 or ≤0.5, and Variable Importance in Projection (VIP) value ≥1.

### Transcriptome Profiling

RNA sequencing was performed by Chi BioTech Co., Ltd. (Shenzhen, China). Briefly, total RNA was extracted using the TRIzol reagent (Ambion, Inc., Austin, TX, USA). RNA libraries were prepared according to the previously described protocol (19). After the library was constructed, Qubit 3.0 was used for preliminary quantification, and then the library was diluted to 1 ng/μl. The Agilent 2100 bioanalyzer was used to detect the insert size of the library. After confirming the insert size met expectations, the Bio-RAD CFX 96 fluorescent quantitative PCR instrument and Bio-RAD KIT iQ SYBR GRN were used to perform Q-PCR. This allowed accurate quantification of the effective concentration of the library (effective concentration of the library > 10 nM). The qualified library was sequenced on the Illumina platform. The sequencing strategy was PE150. The raw reads sequenced from the illumina platform were processed to obtain high-quality sequences (Clean Reads) through the process of removing low-quality sequences and removing connector contamination. All subsequent analyses were based on Clean Reads. Align the filtered sequencing sequence of each sample with the reference genome (Sscrofa11.1) to locate it to the genome by HISAT2 software. Use Fragments per Kilobase per Millon Mapped Fragments (FPKM) to represent gene expression by DESeq2 software. Differential gene screening conditions were: |log2Foldchange|>1, and *P*-value < 0.05. The sequencing results are in Supplementary File 1.

### RNA Extraction and Real-Time qPCR

Total RNA from muscle tissues were isolated with a total RNA extraction kit (OMEGA, USA; Genstar, Beijing, China). Total RNA was reverse transcribed into cDNA using PCR conditions of 95°C for 3 min, followed by 40 cycles of 95°C for 10 s, 60°C for 1 min, and 72°C for 10 s. Primer sequence of qPCR is in Table 2. Cycle threshold values were collected and normalized to that of β-actin.

**Table 2:** qPCR primer sequence.

**Gene name**	**Primer sequence**
Actin F:	CTGGCATTGTCATGGACTCT
Actin R:	GCGATGATCTTGATCTTCAT
FASN F:	ACACCTTCGTGCTGGCCTAC
FASN R:	ATGTCGGTGAACTGCTGCAC
SREBP1 F:	GTGCTGGCGGAGGTCTAGGT
SREBP1 R:	AGGAAGAAGCGGGTCAGAAAG
AKR1B1 F:	AAGGAGCACAGTTCCAAGCAGTCA
AKR1B1 R:	CCCGAAGAGCACTACCTGTAGATT

### Statistics

All data were presented as the means ± SD. Statistical analysis was performed using the unpaired two-tailed *t*-test. The differences were considered statistically significant if *P* < 0.05 (^*^) or *P* < 0.01 (^**^).

## Results

### The IMF Content and Muscle Fiber Type of Luchuan and Duroc Pigs

**Figure 1:**
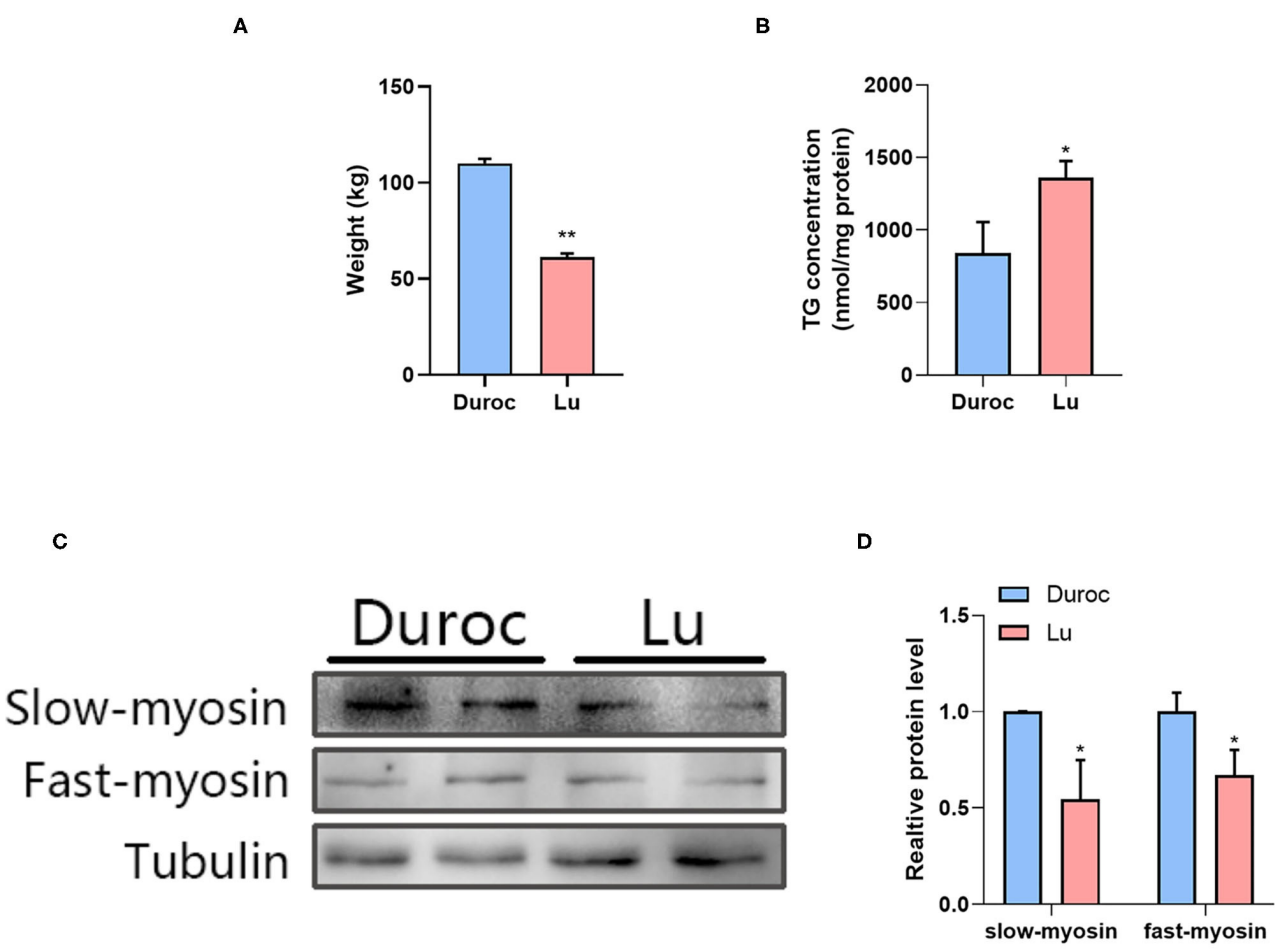
The difference in IMF content and muscle fiber type between Luchuan and Duroc pigs. **(A)** Body weight. **(B)** Muscle triglyceride levels. **(C,D)** Western blot of type I and II muscle fibers and its quantification. The data are expressed as mean ± SD. ^*^P < 0.05, ^**^P < 0.01.

First, we measured the weight of pigs of the same age and found that the body weight of Luchuan pigs was significantly lower than that of Duroc pigs (Figure 1A). In order to understand the difference of IMF content between the two pig breeds, we first determined the fat content in the longissimus dorsi of Luchuan (Lu) and Duroc (Du) pigs. The results show that the triglyceride (TG) content of Luchuan pigs was significantly higher than that of Duroc pigs (Figure 1B). We then performed western blotting on the muscle tissue samples of two pigs to detect the expressions of type I and II muscle fiber. The results showed that the expression levels of type I (slow myosin) and II (fast myosin) muscle fibers were lower in Luchuan pigs than in Duroc pigs (Figures 1C,D).

### Muscle Lipid Composition and Correlation Analysis

**Figure 2:**
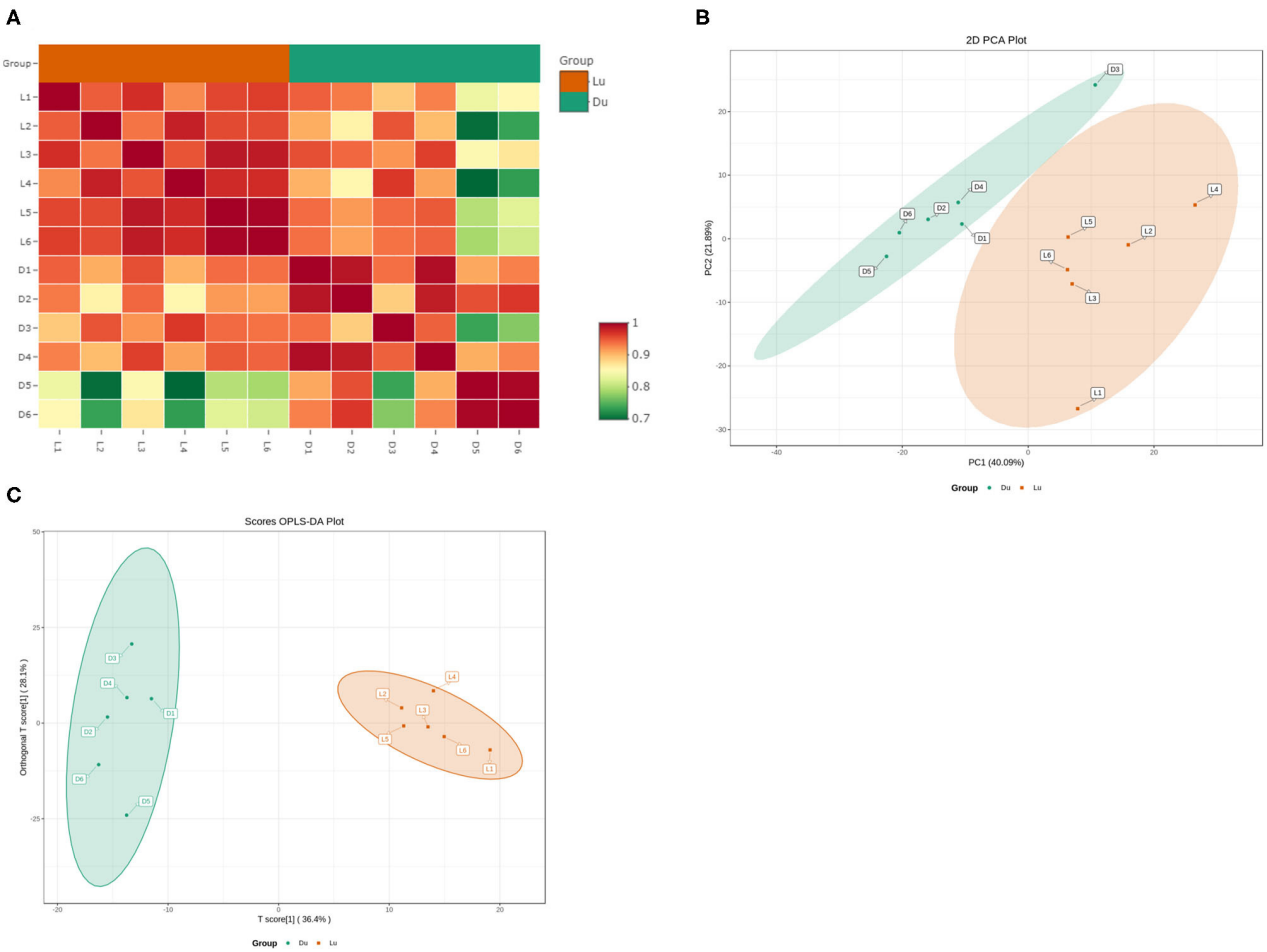
Muscle lipid composition and correlation. **(A)** Lipidomics correlation analysis. **(B)** Principal component analysis (PCA), and **(C)** orthogonal partial least squares discriminant analysis (OPLS-DA) of muscle tissue from six Luchuan and Duroc pigs.

Using the UPLC-MS/MS platform, we extracted lipids from six Luchuan and Duroc pigs. A total of 605 lipids were detected in 23 categories (Table 3). These lipids included 211 triglycerides (TG), 95 phosphatidylcholines (PC), 66 phosphatidylethanolamines (PE), 46 diglycerides (DG), and 37 ceramides (CAR). Correlation analysis showed strong correlation within groups, and that the correlation between the groups was slightly lower, which was consistent with our expectations (Figure 2A). Principal component analysis (PCA) revealed a clear grouping of the two pig breeds in the first two components accounting for 61.89% of the variation (Figure 2B). Orthogonal partial least squares discriminant analysis (OPLS-DA) showed that the difference within group was small, but the difference between the Luchuan and Duroc groups was striking, distinguishing the two pig breeds well (Figure 2C).

**Table 3:** Types and amounts of lipids detected.

**Abbreviation**		**Amount**	**Lipid type**
TG		211	Triglycerides
SM		27	Sphingomyelin
PS		10	Phosphatidylserine
PI		2	Phosphatidylinositol
PG		7	Phosphatidylglycerol
PA		5	Phosphatidic acid
MG		3	Monoglyceride
LPE		10	Lysophosphatidylethanolamine
FFA		29	Free fatty acid
EI		10	Eicosanoid
DG		46	Diglyceride
COQ		2	Coenzyme Q
CAR		37	Acyl Carnitine
PC	PC	63	Phosphatidylcholine
	PC-O	32	Alkyl-acyl-phosphatidylcholines
LPC	LPC	17	Lysophosphatidylcholine
	LPC-O	6	Lysoalkyl-acylphosphatidylcholines
PE	PE	48	Phosphatidylethanolamine
	PE-P	18	Phosphatidylethanolamine-glycerophospholipids
Cer	Cerp	7	Ceramide phosphates
	Cer	8	Ceramide
	Cerm	5	Monohydroxyceramide
	Cer-Hex	2	Hexosyl-ceramides

### Total Lipid Difference Analysis

**Figure 3:**
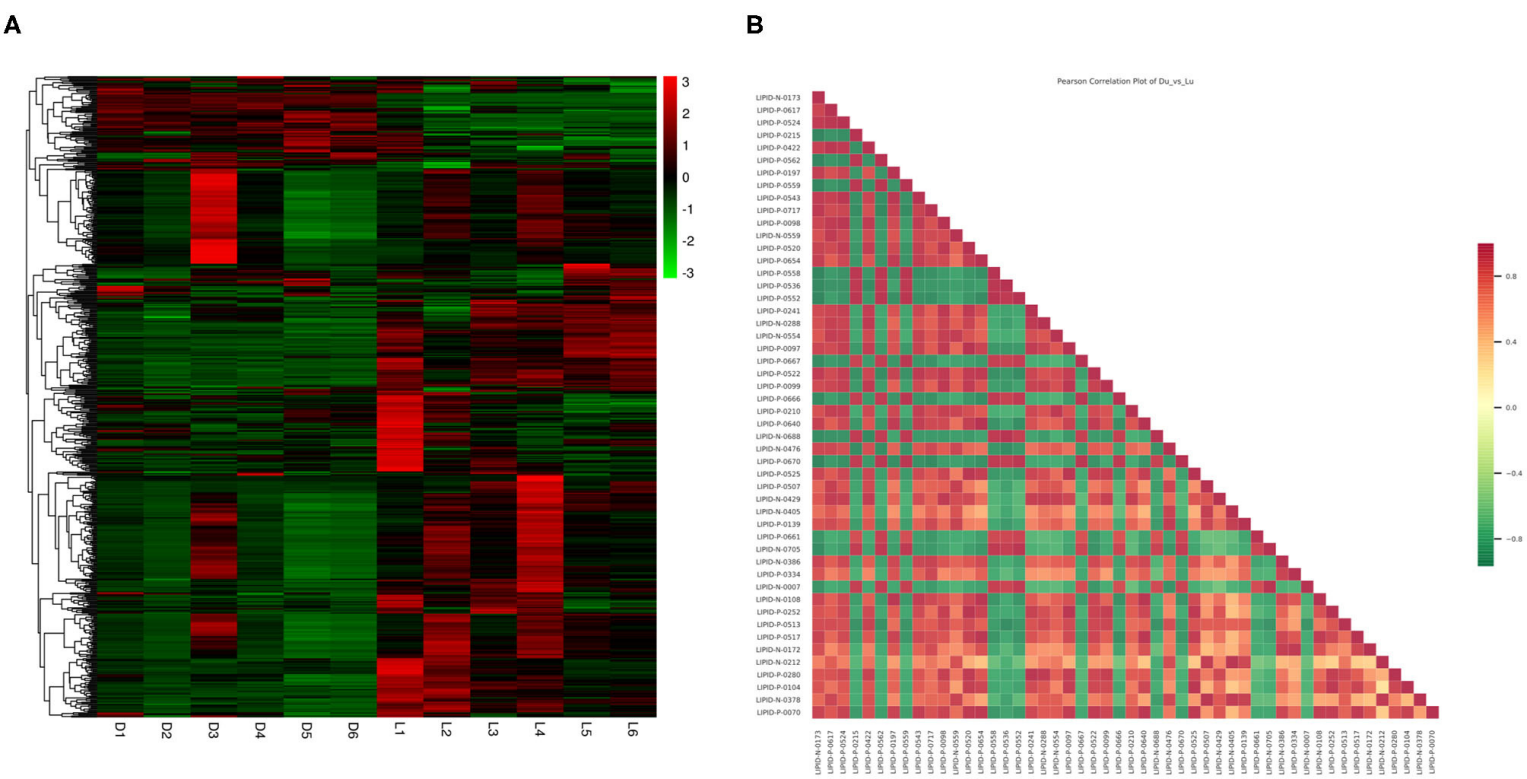
Total lipid difference analysis. **(A)** Overall sample cluster heat map. **(B)** Correlation analysis for significantly different metabolites (top 50 VIP value). Red represents positive correlation, green represents negative correlation.

The Z-score (Supplementary Figure 1) for the expression level of all samples was used to draw an overall sample cluster heat map (Figure 3A). In general, the lipid content of the longissimus dorsi of Duroc pigs was significantly lower than in the Luchuan. Compared with Duroc pigs, Luchuan pigs had 194 lipids upregulated, primarily TGs and PCs (61 and 36, respectively; *P* < 0.05). Only 45 lipids were downregulated, including 18 PCs (Table 4; *P* < 0.05). This also confirms that the IMF content of Luchuan pigs is significantly higher than that of Duroc pigs. Pearson correlation analysis was performed on the significantly different metabolites, mapping the top 50 variable importance in projection (VIP) values, and we found that there were more positively correlated metabolites than negatively correlated metabolites (Figure 3B). This may mean that the accumulation of different lipids promotes further accumulation of lipids.

**Table 4:** Increase and decrease of lipid type and quantity.

**Upregulate lipid type**	**COQ**	**DG**	**TG**	**PA**	**PC**	**PG**	**PE**	**SM**	**LPC**	**LPE**	**Cer**	**CAR**	**FFA**	
Number	1	20	61	5	36	2	27	15	7	2	12	3	3	194
**Downregulate lipid type**	**EI**	**DG**	**PC**	**PS**	**PE**	**SM**	**LPC**	**LPE**	**CAR**	**FFA**				
Number	1	3	18	6	9	2	2	2	1	1				45

### Different Lipid Metabolites Related to Meat Quality and Flavor

**Figure 4:**
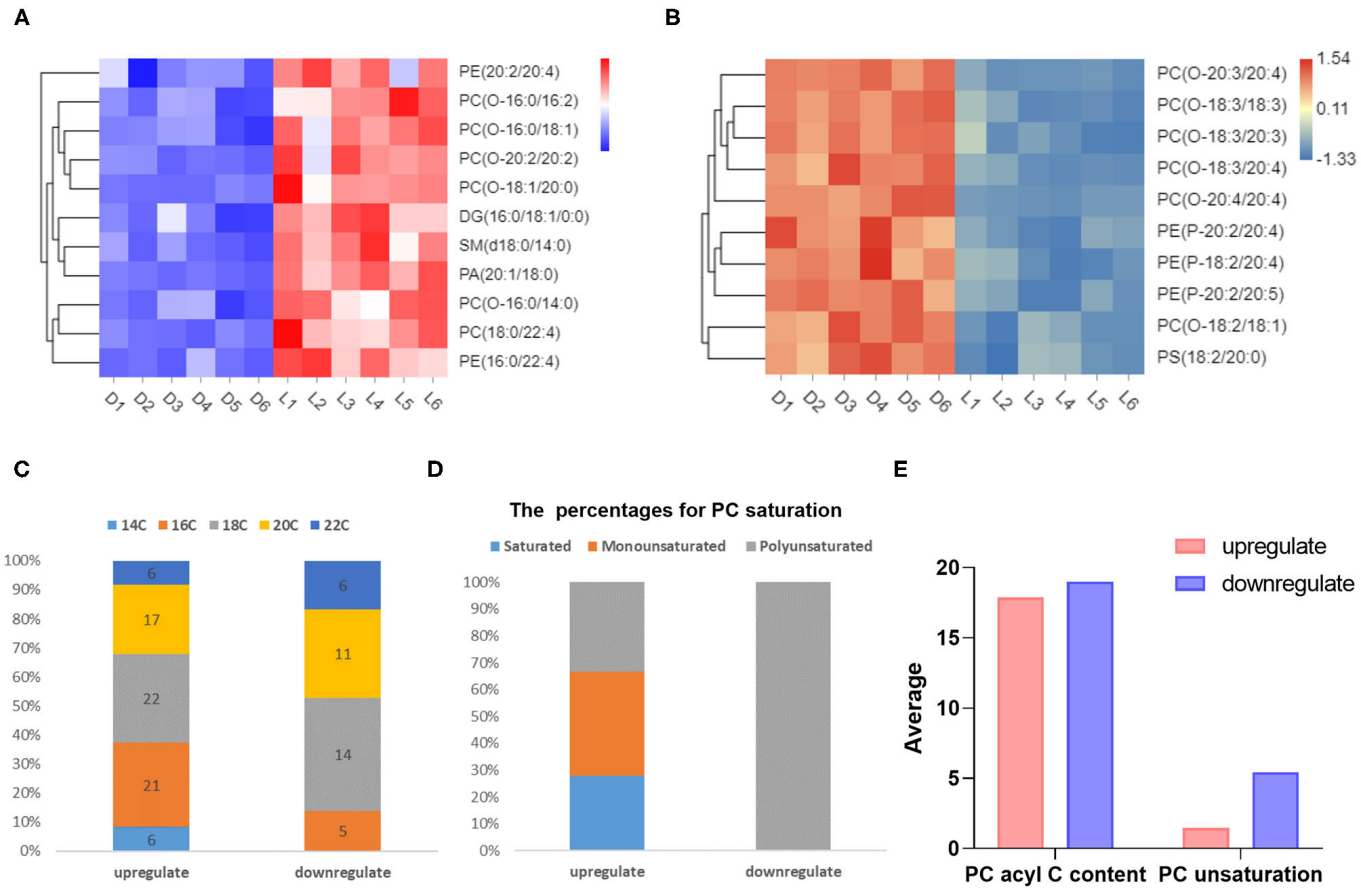
Different metabolites related to meat quality and flavor. **(A)** Top ten upregulated metabolites (*P*-value). **(B)** Top ten downregulated metabolites (*P*-value). **(C)** The number (in the column) of carbons in the alkyl chain of differential metabolites and their percentages. **(D)** Saturation of differential metabolites and their percentages. **(E)** The average number of carbons and saturation in alkyl chains in different metabolites.

TG, DG, and PC are the three main components of lipid in pork, and are closely related to meat flavor (20). We identified 61 species of TGs that were upregulated in Luchuan pigs (*P* < 0.05), and no TG downregulation (Supplementary Figure 2A). Based on fold change, the top ten upregulated metabolites were primarily TGs (Supplementary Figure 2D). There were 20 types of DGs upregulated, and only three were downregulated (Supplementary Figure 2B). This provides an explanation for the superior meat flavor reported in the Luchuan pig compared to the Duroc pig. With respect to PC, there were 36 upregulated and 18 downregulated species (Supplementary Figure 2C). We selected the top ten metabolites with the smallest *P-*value among the upregulated metabolites and prepared a heat map. The heat map showed that the most upregulated metabolites were PCs (Figure 4A). Interestingly, among the top ten downregulated metabolites, the majority were also PCs (Figure 4B). Similarly, looking at the fold change, the results indicated that the majority of metabolites in the top ten downregulated metabolites were PC (Supplementary Figure 2E). This suggests that the PC may be very different between the two pig breeds. Phosphatidylcholine consists of a hydrophilic “head” and a hydrophobic “tail” (21). After statistical analysis of the PC, it was found that the “tail” fatty acid (alkyl) of the down-regulated species contained more carbons (Figure 4C). The fatty acid “tails” of the PCs that were downregulated were all polyunsaturated (Figure 4D). The alkyl chain of the upregulated PCs were shorter, with an average length of 17.89, and the average alkyl chain length of downregulated PCs were 18.94. Furthermore, the up-regulated PCs contained fewer unsaturated bonds, with an average of 0.75 unsaturated bonds per carbon chain, while the downregulated PC had more unsaturated bonds, with an average of 2.72 (Figure 4E).

### Pathway Analysis of Differential Metabolites

**Figure 5:**
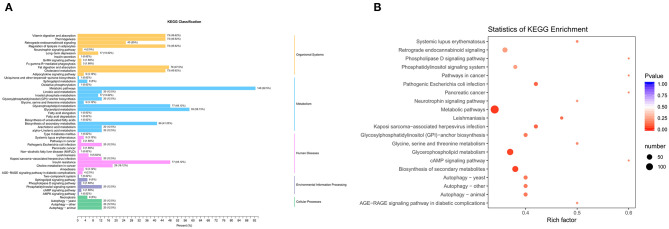
Pathways enriched by differential metabolites. **(A)** KEGG classification. **(B)** Enrichment of KEGG signaling pathway. Rich factor is the ratio of the number of different metabolites in the corresponding pathway to the total number of metabolites detected and annotated by the pathway. The larger the value, the greater the degree of enrichment. The closer the *P*-value is to 0, the more significant the enrichment. The size of the dot in the figure represents the number of significantly different metabolites enriched in the corresponding pathway.

We use the KEGG database (22) to annotate 160 differential metabolites and classify them (Figure 5A). In the organismal systems network, more than 73 differential metabolites (45.62%) were enriched in vitamin digestion and absorption, thermogenesis, regulation of lipolysis in adipocytes, fat digestion and absorption, and cholesterol metabolism. There were 148 metabolites (92.5%) differentially enriched in the metabolic signaling pathway. Among the pathways in human diseases, there were 77 metabolites (48.12%) enriched in insulin resistance pathways. The pathway with the highest degree of enrichment was the metabolic pathway, followed by glycerophospholipid metabolism and biosynthesis of secondary metabolites (Figure 5B).

### Transcriptome Analysis

**Figure 6:**
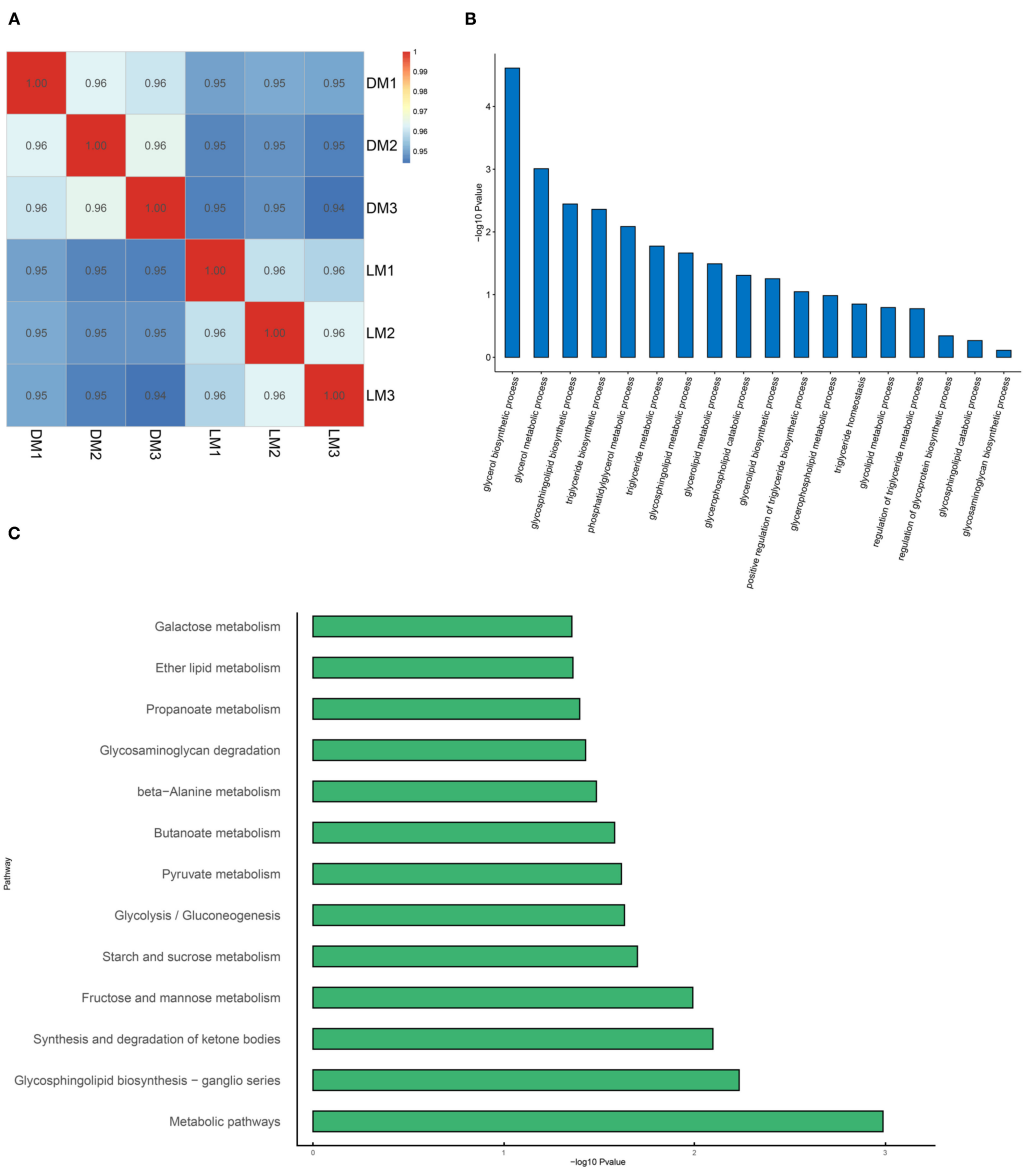
Transcriptome analysis. **(A)** Transcriptome correlation analysis. **(B)** Gene Ontology (GO) enrichment. **(C)** KEGG classification. LM, Luchuan muscle; DM, Duroc muscle.

After transcriptome sequencing of the longissimus dorsi of Luchuan and Duroc pigs, in order to verify the reliability of the results, we analyzed their correlations (Figure 6A). Correlation analysis showed strong correlation within groups, and that the correlation between the groups was slightly lower. After counting the differential genes, we found 471 up-regulated genes, 533 down-regulated genes, and a total of 1,004 differential genes (Table 5). After Gene Ontology (GO) enrichment of differential genes, the biological processes were mainly enriched in processes related to lipid metabolism, among which the difference in glyceride metabolism was the most significant (Figure 6B). After annotating the differential genes with the KEGG pathway, it was found that the most significant pathway is the metabolic pathway, which is consistent with our prediction (Figure 6C).

**Table 5:** The number of differential genes.

**Luchuan vs. Duroc**	**Number**
Up	471
Down	533
Total	1,004

### Joint Analysis of the Transcriptome and Metabolome

**Figure 7:**
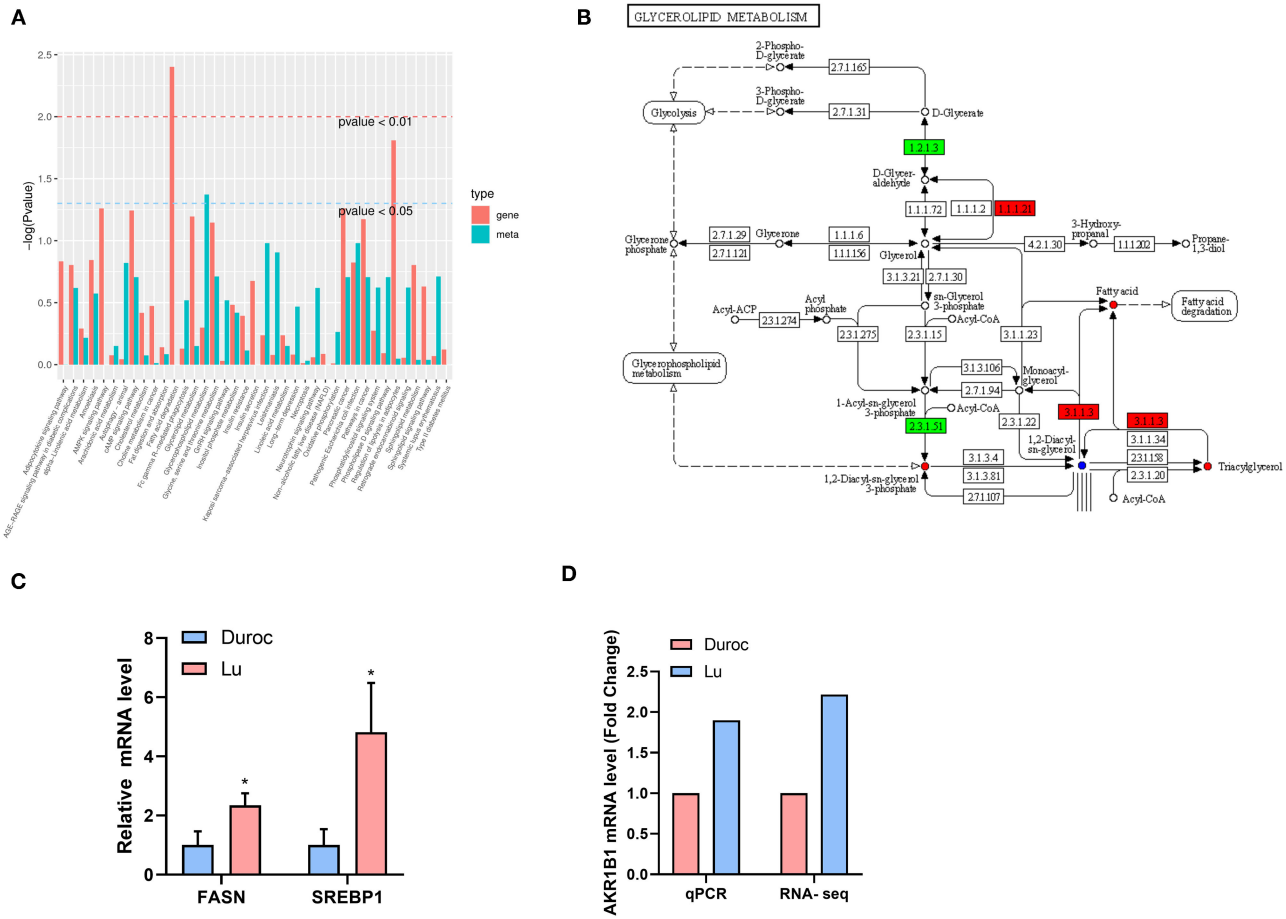
Joint analysis of transcriptome and metabolomics. **(A)** Enrichment of pathway genes and metabolites (red represents the enriched *P-*value of differential genes, and green represents the enriched *P*-value of differential metabolites). **(B)** Differential genes and differential metabolites of the same group of the KEGG pathway (red represents upregulation, green represents downregulation, and blue represents both upregulation and downregulation). **(C)** Lipid synthesis related genes. FASN, fatty acid synthase; SREBP1, Sterol-regulatory element binding protein 1. **(D)** Key genes expression of glycerolipid metabolism pathway. AKR1B1, Aldo-Keto Reductase Family 1 Member B 1. The data are expressed as mean ± SD. *P < 0.05.

Organisms regulate the metabolism of the body by controlling the expression of genes. We conducted a joint analysis of the transcriptome and metabolome data to identify the origin of the metabolic differences between the two pig breeds. The most significant pathways enriched by differential genes are the fat digestion and absorption pathways, followed by regulation of lipolysis in adipocytes (Figure 7A). The results of the enrichment of differential metabolites analysis showed that the biggest difference was in the metabolism of glycerol phosphate, which corresponds to the difference we found in phosphatidylcholine (Figure 4). We used the KEGG pathway diagram, which contains both differential genes and differential metabolites (Figure 7B), and found that both fatty acid and triacylglycerol were increased. This may mean that the high expression of glycerolipid metabolism pathway genes in Luchuan pigs leads to accumulation of more fatty acids and TG. In order to verify our conjecture, we carried out real-time fluorescent quantitative PCR of genes related to lipid synthesis. The results showed that the genes related to lipid synthesis, FASN and SREBP1, were significantly up-regulated in Luchuan pigs (Figure 7C). We also verified the key gene AKR1B1 (Figure 7B -1.1.1.21) in the glycerolipid metabolism pathway and found that it was significantly up-regulated in Luchuan pigs, which is consistent with the transcriptome results (Figure 7D).

## Discussion

In this study, the lipidome of the longissimus dorsi of the obese Chinese native pig breed Luchuan and the western lean pig breed Duroc were analyzed to identify the differences of lipids. Compared to Duroc pigs, Luchuan pigs have higher IMF content, and the degree of unsaturation of phosphatidylcholine were lower. After conducting a joint analysis of the transcriptome, it was found that the differences in lipid deposition may be caused by differences in glycerolipid metabolism pathways.

The transcriptome results showed that the difference of the longissimus dorsi between Luchuan and Duroc pigs was mainly in metabolism, and more importantly in lipid metabolism (Figures 6B,C). This is consistent with the results found in previous studies that there were significant differences in the biosynthesis of unsaturated fatty acids in the skeletal muscle of obese pigs and lean pigs (23). This indicates that the IMF accumulation in obese pigs and lean pigs may be regulated by transcription, which lead to differences in lipid accumulation.

Muscle fiber type and its content are closely related to meat quality. Muscle fiber type can affect the degree of oxidation (24). Studies have shown that fiber size is negatively correlated with muscle oxidation capacity (25). IMF content is positively correlated with tenderness (26) and negatively correlated with fiber diameter (27, 28). IMF directly affects juiciness and flavor, and indirectly affects tenderness (29). Flavor and juiciness were significantly enhanced when IMF levels were over 2.5% (30). The above studies demonstrate the importance of the IMF to meat quality. Compared with obese pig breeds, Duroc pigs have a very low IMF content, while the Chinese native pig breed, Luchuan, is mainly known for its high IMF content. Therefore, studying the difference in lipid metabolism between the Duroc and Luchuan pig can help us better understand the lipid metabolism regulation mechanism and help to improve meat quality.

Phosphatidylcholine (PC) is the main phospholipid in meat that affects meat quality (31). It is a component of the cell membrane and also acts as a reservoir for polyunsaturated fatty acids (PUFA). PUFA has a variety health promoting effects for people with chronic diseases (32). Pork is the main food source of choline in daily life (33). It can relieve symptoms associated with genetically related neurodegenerative diseases (such as Alzheimer's disease) (34). There are also disadvantages associated with polyunsaturated fatty acids in meat products. Some studies have shown that during storage, the oxidation rate of polyunsaturated fatty acids is greater than that of monounsaturated fatty acids and total saturated fatty acids (35). The longissimus dorsi of Luchuan and Duroc pigs contains a lot of PC. The difference between Luchuan and Duroc is that primarily polyunsaturated fatty acids were downregulated in the Luchuan pig. This indicates that the oxidation capacity of Luchuan pork during storage may be lower than that of Duroc pigs, which is more conducive to pork preservation.

Pork is one of the largest meat consumables for humans, and its importance is self-evident. Pig breeds vary greatly in various regions of the world. Luchuan and Duroc, as typical fat and lean pig breeds, are good models for studying fat differences. We analyzed the difference in lipid type and amount in the longissimus dorsi muscle of Luchuan and Duroc pigs, and studied the reasons for this difference. Our research found different lipids related to meat quality and flavor, and identified differential pathways that may affect lipid metabolism using transcriptome analysis. These results provide guidance for improving meat quality.

## Conclusions

Compared with Duroc pigs, Luchuan pigs have higher IMF. The content of flavor-related lipids, such as triglycerides and diglycerides, were also significantly higher in Luchuan pigs than in Duroc pigs. This phenotype may be caused by the higher expression of lipid synthesis-related genes in Luchuan pigs.

## Data Availability Statement

The datasets presented in this study are included in the Supplementary Material, further inquiries can be directed to the corresponding author/s.

## Ethics Statement

The animal study was reviewed and approved by Committee on the Ethics of Animal Experiments of Guangxi University.

## Author Contributions

ZZ, GX, and LZ conceived the project and designed the protocol. ZZ, QL, YS, TP, SL, and YL performed the experiments. ZZ and GX wrote the manuscript. All authors read and approved the final manuscript.

## Conflict of Interest

The authors declare that the research was conducted in the absence of any commercial or financial relationships that could be construed as a potential conflict of interest.

## Supplementary Material

Supplementary Figure 1 Z-score of all samples.

Supplementary Figure 2 **(A)** Overall triglyceride heat map. **(B)** Overall diglyceride heat map. **(C)** Overall phosphatidylcholine heat map. **(D)** Top ten differentially upregulated lipids based on fold change. **(E)** Top ten differentially downregulated lipids based on fold change.

## References

[1] Alfaia CM Lopes PA Madeira MS Pestana JM Coelho D Toldra F. Current feeding strategies to improve pork intramuscular fat content and its nutritional quality. Adv Food Nutr Res. (2019) 89:53–94. 10.1016/bs.afnr.2019.03.006 31351530

[2] Lassaletta L Estelles F Beusen AHW Bouwman L Calvet S van Grinsven HJM. Future global pig production systems according to the shared socioeconomic pathways. Sci Total Environ. (2019) 665:739–51. 10.1016/j.scitotenv.2019.02.079 30790747

[3] Resurreccion AVA. Sensory aspects of consumer choices for meat and meat products. Meat Sci. (2004) 66:11–20. 10.1016/S0309-1740(03)00021-4 22063927

[4] Sun X Young J Liu JH Newman D. Prediction of pork loin quality using online computer vision system and artificial intelligence model. Meat Sci. (2018) 140:72–7. 10.1016/j.meatsci.2018.03.005 29533814

[5] Yu J Zhao P Zheng X Zhou L Wang C Liu JF. Genome-wide detection of selection signatures in duroc revealed candidate genes relating to growth and meat quality. G3. (2020) 10:3765–73. 10.1534/g3.120.401628 32859686 PMC7534417

[6] Wu T Zhang Z Yuan Z Lo LJ Chen J Wang Y. Distinctive genes determine different intramuscular fat and muscle fiber ratios of the longissimus dorsi muscles in Jinhua and landrace pigs. PLoS ONE. (2013) 8:e53181. 10.1371/journal.pone.0053181 23301040 PMC3536781

[7] Ran ML He J Tan JY Yang AQ Li Z Chen B. The complete sequence of the mitochondrial genome of Luchuan pig (Sus scrofa). Mitochondrial DNA A DNA Mapp Seq Anal. (2016) 27:1880–1. 10.3109/19401736.2014.947588 25539161

[8] Hausman GJ Basu U Du M Fernyhough-Culver M Dodson MV. Intermuscular and intramuscular adipose tissues: bad vs. good adipose tissues. Adipocyte. (2014) 3:242–55. 10.4161/adip.28546 26317048 PMC4550684

[9] Katsumata M. Promotion of intramuscular fat accumulation in porcine muscle by nutritional regulation. Anim Sci J. (2011) 82:17–25. 10.1111/j.1740-0929.2010.00844.x 21269355

[10] Sun Y Chen X Qin J Liu S Zhao R Yu T. Comparative analysis of long noncoding RNAs expressed during intramuscular adipocytes adipogenesis in fat-type and lean-type pigs. J Agric Food Chem. (2018) 66:12122–30. 10.1021/acs.jafc.8b04243 30339027

[11] Chen H Wei F Dong X-y Xiang J-q Quek S-y Wang X. Lipidomics in food science. Curr Opin Food Sci. (2017) 16:80–7. 10.1016/j.cofs.2017.08.003

[12] Bourlieu C Cheillan D Blot M Daira P Trauchessec M Ruet S. Polar lipid composition of bioactive dairy co-products buttermilk and butterserum: emphasis on sphingolipid and ceramide isoforms. Food Chem. (2018) 240:67–74. 10.1016/j.foodchem.2017.07.091 28946327

[13] Mi S Shang K Li X Zhang C-H Liu J-Q Huang D-Q. Characterization and discrimination of selected China's domestic pork using an LC-MS-based lipidomics approach. Food Control. (2019) 100:305–14. 10.1016/j.foodcont.2019.02.001

[14] Li J Tang C Zhao Q Yang Y Li F Qin Y. Integrated lipidomics and targeted metabolomics analyses reveal changes in flavor precursors in psoas major muscle of castrated lambs. Food Chem. (2020) 333:127451. 10.1016/j.foodchem.2020.127451 32683255

[15] Tang H Wang X Xu L Ran X Li X Chen L. Establishment of local searching methods for orbitrap-based high throughput metabolomics analysis. Talanta. (2016) 156–7:163–71. 10.1016/j.talanta.2016.04.051 27260449

[16] Qi Y Zhang Z Liu S Aluo Z Zhang L Yu L. Zinc supplementation alleviates lipid and glucose metabolic disorders induced by a high-fat diet. J Agric Food Chem. (2020) 68:5189–200. 10.1021/acs.jafc.0c01103 32290656

[17] Zhang L Qi Y Z AL Liu S Zhang Z Zhou L. Betaine increases mitochondrial content and improves hepatic lipid metabolism. Food Funct. (2019) 10:216–23. 10.1039/C8FO02004C 30534761

[18] Liu S Yang D Yu L Z AL Zhang Z Qi Y. Effects of lycopene on skeletal muscle fiber type and high fat diet induced oxidative stress. J Nutr Biochem. (2020) 87:108523. 10.1016/j.jnutbio.2020.108523 33039582

[19] Luo Z Hu H Liu S Zhang Z Li Y Zhou L. Comprehensive analysis of the translatome reveals the relationship between the translational and transcriptional control in high fat diet-induced liver steatosis. RNA Biol. (2020) 6:1–12. 10.1080/15476286.2020.1827193 32967529 PMC8081042

[20] Huang Y-C Li H-J He Z-F Wang T Qin G. Study on the flavor contribution of phospholipids and triglycerides to pork. Food Sci Biotechnol. (2010) 19:1267–76. 10.1007/s10068-010-0181-0

[21] Rudolphi-Skorska E Filek M Zembala M. The effects of the structure and composition of the hydrophobic parts of phosphatidylcholine-containing systems on phosphatidylcholine oxidation by ozone. J Membr Biol. (2017) 250:493–505. 10.1007/s00232-017-9976-8 28799139 PMC5613038

[22] Kanehisa M Goto S. KEGG: kyoto encyclopedia of genes and genomes. Nucleic Acids Res. (2000) 28:27–30. 10.1093/nar/28.1.27 10592173 PMC102409

[23] Yang Y Liang G Niu G Zhang Y Zhou R Wang Y. Comparative analysis of DNA methylome and transcriptome of skeletal muscle in lean-, obese-, and mini-type pigs. Sci Rep. (2017) 7:39883. 10.1038/srep39883 28045116 PMC5206674

[24] Klont RE Brocks L Fau - Eikelenboom G Eikelenboom G. Muscle fibre type and meat quality. Meat Sci. (2000) 49S1:S219–29. 10.1016/S0309-1740(98)90050-X 22060713

[25] Lee SH Kim JM Ryu YC Ko KS. Effects of morphological characteristics of muscle fibers on porcine growth performance and pork quality. Korean J Food Sci Anim Resour. (2016) 36:583–93. 10.5851/kosfa.2016.36.5.583 27857533 PMC5112420

[26] Renand G Picard B Touraille C Berge P Lepetit J. Relationships between muscle characteristics and meat quality traits of young Charolais bulls. Meat Sci. (2001) 59:49–60. 10.1016/S0309-1740(01)00051-1 22062505

[27] Karlsson AH Klont RE Fernandez XJLPe. Skeletal muscle fibres as factors for pork quality. Livestock Prod Sci. (1999) 60:255–69. 10.1016/S0301-6226(99)00098-6

[28] Estany J Ros-Freixedes R Tor M Pena RN. Triennial growth and development symposium: genetics and breeding for intramuscular fat and oleic acid content in pigs. J Anim Sci. (2017) 95:2261–71. 10.2527/jas2016.1108 28727022

[29] Jeremiah LE Dugan ME Aalhus JL Gibson LL. Assessment of the relationship between chemical components and palatability of major beef muscles and muscle groups. Meat Sci. (2003) 65:1013–9. 10.1016/S0309-1740(02)00309-1 22063683

[30] Fernandez X Monin G Talmant A Mourot J Lebret B. Influence of intramuscular fat content on the quality of pig meat - 2. Consumer acceptability of *M. longissimus* lumborum. Meat Sci. (1999) 53:67–72. 10.1016/S0309-1740(99)00038-8 22062934

[31] Enomoto H Furukawa T Takeda S Hatta H Zaima N. Unique distribution of diacyl-, alkylacyl-, and alkenylacyl-phosphatidylcholine species visualized in pork chop tissues by matrix-assisted laser desorption/ionization-mass spectrometry imaging. Foods. (2020) 9:205. 10.3390/foods9020205 32079116 PMC7073967

[32] Ma X Jiang Z Lai C. Significance of increasing n-3 PUFA content in pork on human health. Crit Rev Food Sci Nutr. (2016) 56:858–70. 10.1080/10408398.2013.850059 26237277

[33] Lewis ED Zhao YY Richard C Bruce HL Jacobs RL Field CJ. Measurement of the abundance of choline and the distribution of choline-containing moieties in meat. Int J Food Sci Nutr. (2015) 66:743–8. 10.3109/09637486.2015.1088942 26401718

[34] Bekdash RA. Neuroprotective effects of choline and other methyl donors. Nutrients. (2019) 11:2995. 10.3390/nu11122995 31817768 PMC6950346

[35] Huang Y He Z Li H Li F Wu Z. Effect of antioxidant on the fatty acid composition and lipid oxidation of intramuscular lipid in pressurized pork. Meat Sci. (2012) 91:137–41. 10.1016/j.meatsci.2012.01.006 22317893

