# Supplementary figures and images for: Lipidomic and Transcriptomic Analysis of the Longissimus Muscle of Luchuan and Duroc Pigs
Source: Front Nutr. 2021 May 7;8:667622. doi: 10.3389/fnut.2021.667622 (PMC8154583; doi:10.3389/fnut.2021.667622)

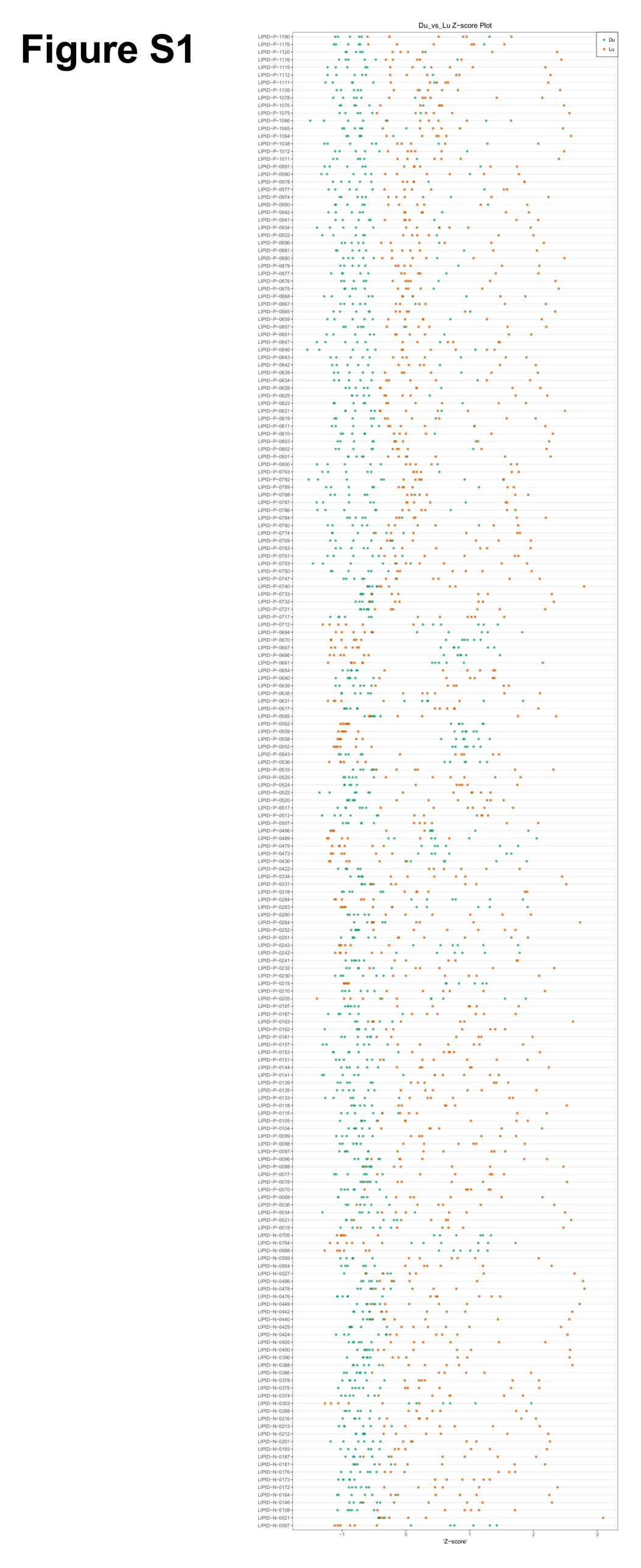

Supplement: Supplementary Figure 1 — Z-score of all samples. [file Image_1.TIF]

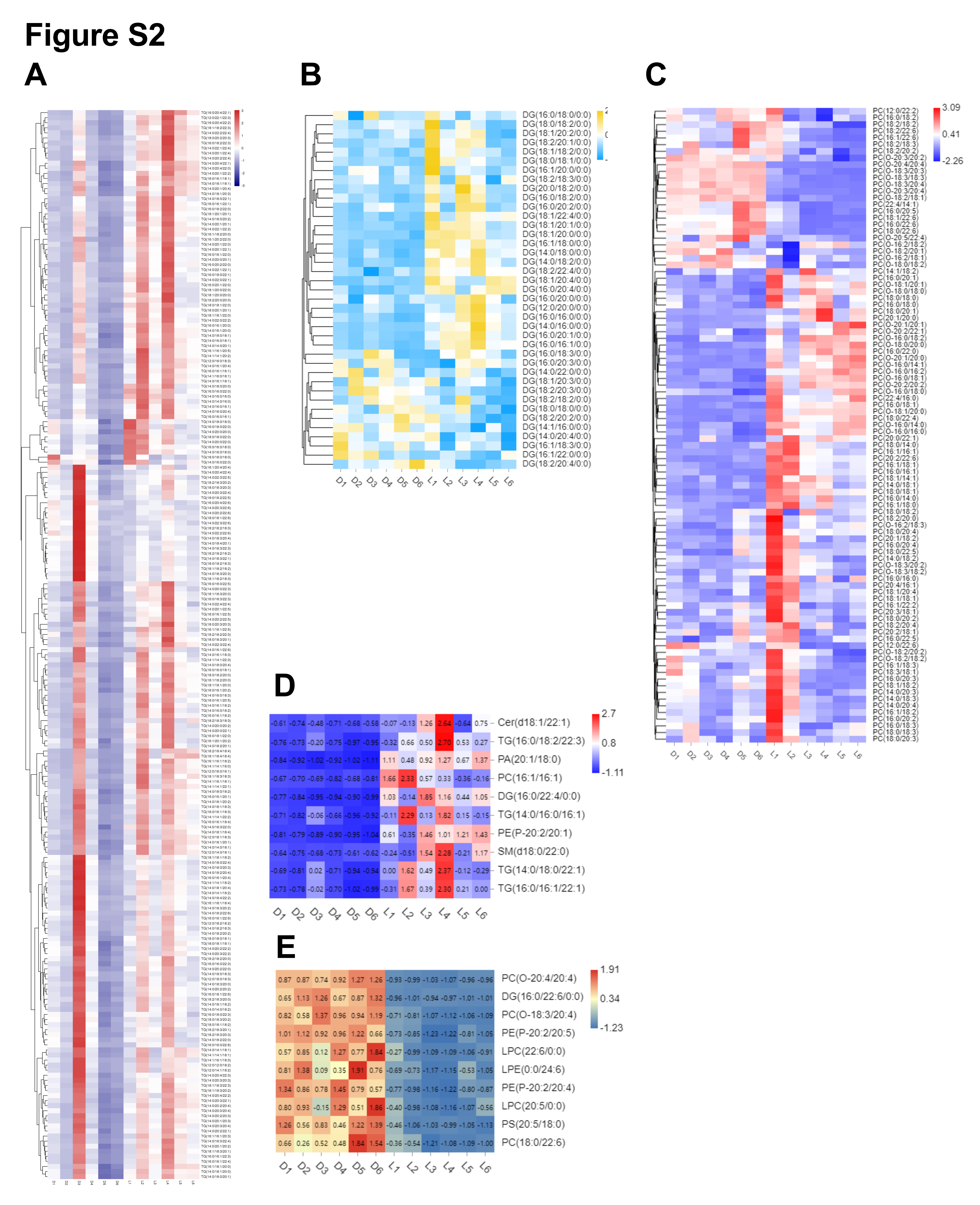

Supplement: Supplementary Figure 2 — (A) Overall triglyceride heat map. (B) Overall diglyceride heat map. (C) Overall phosphatidylcholine heat map. (D) Top ten differentially upregulated lipids based on fold change. (E) Top ten differentially downregulated lipids based on fold change. [file Image_2.TIF]
